# Development of a Portfolio Diet Score and Its Concurrent and Predictive Validity Assessed by a Food Frequency Questionnaire

**DOI:** 10.3390/nu13082850

**Published:** 2021-08-19

**Authors:** Andrea J. Glenn, Beatrice A. Boucher, Chloe C. Kavcic, Tauseef A. Khan, Melanie Paquette, Cyril W. C. Kendall, Anthony J. Hanley, David J. A. Jenkins, John L. Sievenpiper

**Affiliations:** 1 Department of Nutritional Sciences, Temerty Faculty of Medicine, University of Toronto, Toronto, ON M5S 1A8, Canada; andrea.glenn@utoronto.ca (A.J.G.); beatrice.boucher@utoronto.ca (B.A.B.); tauseef.khan@utoronto.ca (T.A.K.); melanie.paquette@utoronto.ca (M.P.); cyril.kendall@utoronto.ca (C.W.C.K.); anthony.hanley@utoronto.ca (A.J.H.); david.jenkins@utoronto.ca (D.J.A.J.); 2 Clinical Nutrition and Risk Factor Modification Center, St. Michael’s Hospital, Toronto, ON M5C 2T2, Canada; 3 Toronto 3D Knowledge Synthesis and Clinical Trials Unit, St. Michael’s Hospital, Toronto, ON M5C 2T2, Canada; 4 School of Nutrition, Ryerson University, Toronto, ON M5B 2K3, Canada; chloe.kavcic@ryerson.ca; 5 College of Pharmacy and Nutrition, University of Saskatchewan, Saskatoon, SK S7N 5E5, Canada; 6 Department of Medicine, Dalla Lana School of Public Health, University of Toronto, Toronto, ON M5T 3M7, Canada; 7 Leadership Sinai Centre for Diabetes, Mount Sinai Hospital, Toronto, ON M5G 1X5, Canada; 8 Department of Medicine, Temerty Faculty of Medicine, University of Toronto, Toronto, ON M5S 1A8, Canada; 9 Li Ka Shing Knowledge Institute, St. Michael’s Hospital, Toronto, ON M5B 1A6, Canada; 10 Division of Endocrinology and Metabolism, Department of Medicine, St. Michael’s Hospital, Toronto, ON M5C 2T2, Canada

**Keywords:** portfolio diet, validity, diet index, dietary pattern

## Abstract

The Portfolio Diet, a plant-based portfolio of cholesterol-lowering foods, has been shown to reduce low-density lipoprotein cholesterol (LDL-C), and other cardiovascular risk factors, in randomized controlled trials (RCTs). It is not known if these beneficial effects translate to a lower incidence OF cardiovascular disease (CVD). To support examinations between Portfolio Diet adherence and disease, a Portfolio Diet score (PDS) was developed and its predictive and concurrent validity was assessed within the Toronto Healthy Diet Study, a six-month RCT in overweight adults. Predictive validity was assessed using change in the PDS measured by food frequency questionnaire (FFQ) and concomitant change in LDL-C from baseline to six months using multiple linear regression, adjusted for potential confounders (*n* = 652). Concurrent validity was assessed in a subset of participants (*n* = 50) who completed the FFQ and a 7-day diet record (7DDR) at baseline. The PDS determined from each diet assessment method was used to derive correlation coefficients and Bland–Altman plots to assess the between-method agreement. The change in PDS was inversely associated with change in LDL-C (β coefficients: −0.01 mmol/L (95% confidence intervals (CIs): −0.02, −0.002; *p* = 0.02). The correlation between the PDS from the FFQ and 7DDR was 0.69 (95% CIs: 0.48, 0.85). The Bland–Altman plot showed reasonable agreement between the score from the FFQ and 7DDR. These findings indicate predictive validity of the PDS with lower LDL-C, and reasonable concurrent validity of the PDS as assessed by an FFQ against a 7DDR.

## 1. Introduction

The Portfolio Diet, or Dietary Portfolio, is a plant-based dietary pattern that was developed in the early 2000s as a “portfolio” of four cholesterol-lowering foods and nutrients (plant protein, viscous fiber, nuts and phytosterols) [1] that each have Health Canada, U.S Food and Drug Administration, and/or European Food Safety Authority approved health claims for cholesterol-lowering or cardiovascular disease (CVD) risk reduction [2,3,4,5,6,7,8,9]. The Portfolio Diet is low in saturated fat and cholesterol (National Cholesterol Education Program (NCEP) Step II diet) [10], with four food components added to the diet (based on a 2000 kcal diet): 50 g plant protein from soy products or dietary pulses such as beans, peas, chickpeas, and lentils; 20 g viscous soluble fiber from oats, barley, psyllium, eggplant, okra, and certain fruit; 45 g nuts (tree nuts or peanuts); and 2 g phytosterols originally provided as an enriched margarine [1,11,12,13,14]. A fifth component was later added to the diet, which includes plant-based monounsaturated fats (MUFAs) in the form of olive, canola, high oleic sunflower oils, or avocados (45 g/day) to further improve blood lipids, including high-density lipoprotein (HDL) cholesterol [15]. Early findings from a metabolically controlled randomized controlled trial (RCT) showed that the low density-lipoprotein cholesterol (LDL-C) lowering effect of the Portfolio Diet was similar to 20 mg lovastatin (−28.6% vs. −30.9%) [12]. A recent systematic review and meta-analysis of metabolically controlled and ad libitum trials showed that the Portfolio Diet significantly lowered LDL-C by 17% (27% in the intended combination with an NCEP Step II diet), among several other CVD risk factors, including non-HDL-C, apolipoprotein B and C-reactive protein [16].

It is currently not known if the LDL-C lowering effect of the Portfolio Diet translates into lower risk of clinical CVD events. Conducting a long-term RCT with CVD as the primary outcome would be preferable; however, this type of trial will be challenging to conduct from a logistical and funding perspective. Therefore, analyses of observational studies, such as prospective cohort studies, may help assess the long-term effectiveness of the Portfolio Diet.

A priori dietary patterns that are based upon nutrition knowledge of a diet shown to be healthful, such as the Portfolio Diet, are typically assessed with disease outcomes in prospective cohorts through the creation of a diet index or score. However, adherence to the Portfolio Diet has not been assessed in these study designs, aside from two recent studies by our group [17,18], and a diet index/scoring system for this diet has not been formally described or evaluated in terms of its validity. A Portfolio Diet score (PDS) will be particularly useful applied to food frequency questionnaires (FFQs), as they are the most common diet assessment tools used in population-based observational studies [19], including prospective cohort studies. The objectives of this study were to describe the development of a PDS and to assess the predictive and concurrent validity of our PDS as measured by an FFQ. Predictive validity was assessed against LDL-C and concurrent validity was assessed between the PDS from the FFQ and 7-day diet records as the reference method.

## 2. Methods

### 2.1. Study Population

**Figure 1:**
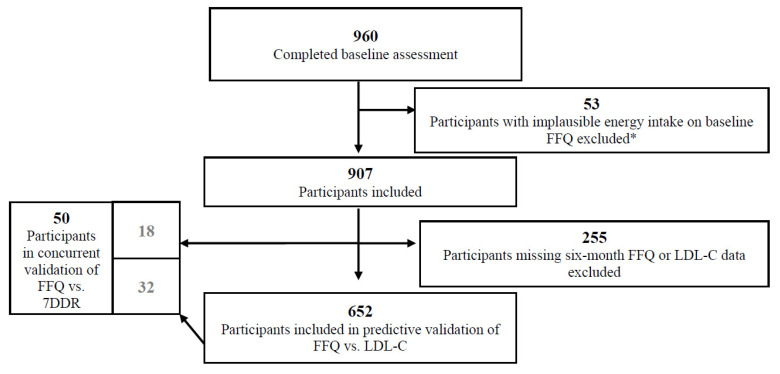
Flowchart for the validation study sample, Toronto Healthy Diet Study. Abbreviations: FFQ, food frequency questionnaire; LDL-C, low-density lipoprotein cholesterol; 7DDR, 7-day diet record. * Women who reported a total daily energy intake of <500 or >4000 kcal, and men with a <800 or >5000 kcal on the baseline FFQ were excluded from the analysis.

The development and validation of the PDS was conducted within the Toronto Healthy Diet Study (THDS). The THDS was a six-month population-based RCT assessing the effects of a healthy diet intervention in overweight (BMI > 25 kg/m^2^) adults in Toronto, with 960 participants completing a baseline assessment (including FFQ, blood sample and other questionnaires). Participants were recruited between 2005 and 2009 using public advertisements (e.g., on transit). Participants were randomized as households (households of one or more), and all members of the same household received the same diet (*n* = 94 two-member households; *n* = 3 three-member households). Women who reported a total daily energy intake of <500 or >4000 kcal, and men with a <800 or >5000 kcal on the baseline FFQ were excluded from the analysis. Participants with missing LDL-C or FFQ assessment at six months were also excluded from the predictive validation analysis. A total of 652 participants were included in the predictive validity analysis and 50 in the concurrent validity analysis (Figure 1). Thirty-two participants were included (i.e., had overlap) in both the predictive and concurrent validity analyses (Figure 1).

### 2.2. Study Design 

Participants completed an FFQ, other questionnaires, and provided fasting blood, anthropometric, and blood pressure measurements at baseline and six months. All visits took place at St. Michael’s Hospital in Toronto. Research ethics boards of the University of Toronto and St. Michael’s Hospital approved the protocol and all participants provided written informed consent. The trial was registered with ClinicalTrials.gov (NCT00516620). Further information on the trial has been previously published [20].

### 2.3. Nutrition Intervention 

The trial was a six-month intervention comparing the control group (general advice to follow Canada’s Food Guide, either the 1992 or 2007 edition depending on recruitment date [21]) to those randomized to receive one of three diet interventions: (1) dietary advice from a Registered Dietitian (RD) to follow healthy eating principles (such as increased fruit and vegetable intake, whole grain intake, etc.), (2) weekly food baskets reflecting the dietary advice, or (3) dietary advice from an RD and weekly food baskets.

### 2.4. Dietary Assessment

#### 2.4.1. Food Frequency Questionnaire

A semi-quantitative FFQ asking about usual consumption over the past month was self-administered by study participants at baseline and six months [20]. Serving sizes were preassigned and most food items had 8 or 9 continuous frequency responses ranging from ‘never’ to ‘2 or more’ or ‘4 or more’ per day. The original validated FFQ by Willet et al. [22,23] was expanded to 184 food items to improve the assessment of viscous fiber, nuts, whole grains, glycemic index, and caffeine. Modifications included adding 26 food items, including six items relevant to the Portfolio Diet (cooked oat bran, barley, eggplant, almond butter, almonds, and walnuts), and changes to expand or clarify inclusions (e.g., raspberries and blackberries were added to the item for strawberries). Coding rules for open-ended questions about the usual type and brand of cold breakfast cereals (e.g., Bran Buds), cooking oils, margarine, and multivitamin supplements were also revised for the Canadian market. Supplement items included dose and duration of use for multivitamins and 10 individual nutrients, as well as a checklist of 10 other supplements (including Metamucil) to identify those taken currently at least once a week. The FFQs were mailed to participants and reviewed for completion by study RDs at clinic visits or via telephone. FFQ analysis to derive food and nutrient intakes was based on USDA values and provided by the Nutrition Department at Harvard T.H. Chan School of Public Health.

#### 2.4.2. Diet Records

A subsample of 50 participants also completed a 7-day diet record (7DDR) on consecutive days at baseline. A 7DDR booklet and instructions were mailed to participants after completing the FFQ. These participants were some of the first to be recruited into the study, and they completed both FFQs and 7DDR before the latter were discontinued due to high respondent burden. Study RDs reviewed the 7DDRs for completeness with participants at the clinic or over the telephone. The 7DDRs were entered into and analyzed using ESHA Research Food Processor SQL: Nutrition Analysis and Fitness Program (Copyright 2012, ESHA Research).

### 2.5. Development of the Portfolio Diet Score

Development of the PDS followed a similar approach to the well-known Dietary Approaches to Stop Hypertension (DASH) diet score [24], which originated from RCT evidence and then later applied to other study designs. We considered several factors during the development of our PDS, including:

#### 2.5.1. Using Dietary Recommendations from the RCTs or Population-Specific Cut-Offs

In the Portfolio Diet RCTs, participants were provided with foods and/or advised to consume specific foods to meet the following recommendations: 50 g of plant protein, 20 g of viscous fiber, 45 g of nuts, 2 g of phytosterols, 45 g of MUFAs and ≤7% total energy from saturated fat and ≤200 mg dietary cholesterol [16]. Using these specific recommendations as adherence cut points appeared ideal at first; however, it is important to consider the context in which we are applying the created score. In the current study, the PDS is being applied to data from a healthy diet intervention trial, with plans to apply the PDS to other population level studies, such as large prospective cohorts, in the future. As these study populations were not advised to follow the Portfolio Diet, it is likely that intake of certain recommended foods or nutrients will remain below the desired cut-off level (e.g., 50 g of plant protein) for many participants and therefore, these cut-off levels will have reduced discriminating power between groups. The ability to discriminate between groups of individuals is necessary to assess disease outcome associations [25,26]. It is also important to consider that even if a population does not meet the Portfolio Diet specific recommendations, beneficial changes may still occur, as reported in the Jenkins et al., 2011 dietary advice RCT where significant clinically relevant reductions (−15%) in LDL-C were seen, although participants were only 43% adherent on average [14]. Given these considerations, we used population-specific cut-offs rather than the RCT recommendations to create the PDS. To compare Portfolio Diet adherence in the current study against RCT recommendations, we estimated mean intake of PDS components by quintiles using FFQ data at baseline and six months (*n* = 652).

#### 2.5.2. Using a Nutrient or Food-Based Score

The Portfolio Diet recommendations listed above were initially conceptualized as nutrients (g/day); however, this is not how the dietary advice is provided. Individuals, including the participants in the Portfolio Diet RCTs [16], receive education to include or limit specific foods. Food-based scores (assessed as servings per day, for example), rather than nutrient-based scores, are also generally easier to interpret and to apply to multiple diet assessment settings and tools (FFQs, diet records, etc.) Food-based scores do not require compositional databases to include the specific nutrients of interest or to be updated for changes in the food supply or changes in analytical methods. Food-based approaches are also more in line with current dietary and clinical practice guidelines that focus on foods and dietary patterns, rather than on macro and micronutrient recommendations [27,28,29,30].

The one exception to a food-based approach was for phytosterols, where we estimated intake in mg/day. In the Portfolio Diet RCTs, phytosterols were provided through supplemented foods (originally as an enriched margarine). As these products were not available to participants in the current study and may not be applicable to other population-based studies, we assessed total dietary intake of phytosterols, which are present in all plant foods. In anticipation of some overlap of phytosterol food sources with the other plant-based components recommended in the diet (plant protein, viscous fiber, nuts and MUFAs), we assessed interitem correlations between each of the score components (*n* = 652).

#### 2.5.3. Choice of Quantiles

The choice of quantiles to use when constructing population-specific cut-offs in diet scores is subjective and various quantiles have been used in the literature. The most common approaches have used medians (assigning 0 points for below and 1 point for above), such as in several Mediterranean or Nordic diet scores [31,32,33], as well as quintiles (assigning 1 and 5 points across quintiles), such as in a DASH or the overall, healthy and unhealthy plant-based diet indices [24,34]. Our a priori plan was to use the quintile-based approach; however, we conducted a sensitivity analysis to compare the association of the PDS with LDL-C using the median approach (score range 0–6) and the quintile approach (score range 6–30) using the same methods described below for the predictive validity analysis (*n* = 652) to assess how the two cut-off approaches may impact the results. Medians or quintiles of intake in our study were assessed separately for men and women.

#### 2.5.4. Weight of Each Diet Component

A limitation of diet scores reported in the literature is that the weight of each score component is not taken into account [25,35,36,37]. This lack of reporting in previous studies may be because the relative effect of different components on the outcome of interest is unknown, and the weight is likely different for various health outcomes [25,35,36,37]. The researchers who created the DASH diet index reported that it is difficult to specify the contribution of each food group contained in their score to CVD risk [24]. However, to create the PDS, we explored weighting the score components differently based on past LDL-C research. Previous evidence showed that plant protein, viscous fiber, nuts and phytosterols can lower LDL-C by ~5% each [38,39,40,41,42,43], whereas the low saturated fat and cholesterol component lowered LDL-C by ~10% in the Portfolio Diet trials [16]. Consequently, we conducted a sensitivity analysis where we provided double weight (i.e., points) to the low saturated fat/cholesterol component to explore if there were differences in the PDS association with LDL-C, using the same methods described below for the predictive validity analysis (*n* = 652).

### 2.6. Calculating the PDS

Food items relevant to the Portfolio Diet were identified in the FFQ and categorized into the six Portfolio Diet components: plant protein, viscous fiber, nuts, phytosterols, monounsaturated fats (MUFAs), and saturated fat/cholesterol sources. Since food items had pre-assigned serving sizes, the frequency category reported for each food item was directly converted to servings/day (i.e., “2–4 times per week” was 0.43 servings/day). For all components, servings/day were summed over all consumed food items in each component. Phytosterols required an additional step to convert servings/day to mg/day using serving size weights provided by the Nutrition Department at the Harvard T.H. Chan School of Public Health, and food content values (mg/100 mg) in our phytosterol database described below.

More points were given to participants with higher intakes of foods recommended in the Portfolio Diet, whereas less points were given to participants with higher intakes of foods to be limited in the Portfolio Diet. These points were given for each of the six components by splitting them into sex-specific quintiles: those in the highest quintile of foods recommended (such as nuts) received five points and those in the lowest quintile received one point. Reverse scoring was used for those foods to be limited (such as foods high in saturated fat), where those with the highest intake (quintile 5) received one point, and those with the lowest intake (quintile 1) received five points. The total points were then added for each participant, resulting in a possible score range between 6 and 30, with higher scores indicating higher adherence to the Portfolio Diet. A similar approach was utilized when creating the score using medians, where those above the median for foods recommended in the diet received 1 point, and those below received 0 points (score range 0–6).

We then used the 7DDR data to create the PDS. To obtain comparable data from the FFQ and 7DDR, the unique foods from the 7DDRs were matched to the six components of our PDS. Serving sizes from the 7DDR were converted to match those included on the FFQ. The food group components created from the 7DDRs were then scored in the same way as discussed above to create the PDS in the FFQ.

### 2.7. Phytosterol Database

Phytosterols were the only score component based on mg/day. The FFQ compositional database from the Harvard T.H. Chan School of Public Health used in this study and the 7DDR compositional database (ESHA Research Food Processor SQL: Nutrition Analysis and Fitness Program (Copyright 2012, ESHA Research)) did not have phytosterols available as a nutrient variable. Therefore, we developed a phytosterol database (mg/100 g) based on literature values and existing databases and created recipes to match the food items in the FFQ and 7DDRs. Several data sources were used, including the Finnish Food Composition Database [44], the European Prospective Cohort into Cancer (EPIC) Netherlands database [45], the United States Department of Agriculture (USDA) [46], and other literature [47,48,49,50,51]. Original analytical values were used when available, as well as imputed values for unavailable foods where similar foods were available, and calculated values for recipes using foods with available and imputed values. Recipes were analyzed using the ESHA program. Since the content data of individual phytosterols were not consistently available for all foods, we assessed total phytosterols summing values for the 5–7 most frequently occurring phytosterols in foods reported in the literature (sitosterol, campesterol, stigmasterol, 5-avenasterol, brassicasterol, sitostanol and campestanol).

### 2.8. LDL-C Assessment

Blood samples were collected after a 12-h overnight fast at baseline and six-month clinic visits and analyzed at the St. Michael’s Hospital Core Laboratory, Toronto. LDL-C was calculated in mmol/L by the method of Friedewald et al. [52]

### 2.9. Covariate Assessment

Measurements of demographic, socioeconomic, medical and lifestyle variables, including smoking and medical history, were obtained through structured self-administered questionnaires and reviewed by the study RDs. Physical activity was assessed using the short-form International Physical Activity Questionnaire (IPAQ) [53]. Participants’ body weight and height were measured without shoes and in light indoor clothing at baseline.

### 2.10. Statistical Analysis 

Descriptive analysis to compare the baseline characteristics (including intake assessed by baseline FFQ) of those in the predictive validation group using FFQ and LDL-C data to the concurrent validation subset that completed the FFQ and 7DDR was conducted by using Student *t*-test for continuous variables and chi-square test for categorical variables.

For the predictive validity analysis, we used multiple linear regression and Pearson correlation coefficients to examine the association and correlation of change in the PDS with concomitant change in LDL-C, and logistic regression to examine the odds of having a 5% reduction in LDL-C with change in the PDS. In all models, we used robust variance estimates to account for intra-cluster correlations (*n* = 94 two-member households; *n* = 3 three-member households) to account for households randomized together in the main trial. We adjusted for the following covariates in the fully adjusted model: sex (male/female), age (continuous), baseline LDL-C (continuous), energy intake (continuous), use of cholesterol-lowering medications (yes/no), ethnicity (Caucasian/Asian/African and Caribbean/Other/Unknown), BMI (continuous), smoking status (current/never and past), family history of CVD (yes/no), education (high school or less/undergraduate and college/graduate), physical activity level (low/medium/high) and intervention group (control/intervention). These same methods were repeated in our sensitivity analyses to assess the effect of median and quintile cut-offs, and weighting of the saturated fat/cholesterol component during the PDS development.

For the concurrent validation analyses, we calculated Pearson correlation coefficients between the PDS from the FFQ and the PDS from the 7DDR, with and without energy adjustment (using the residual method) as absolute score (6–30) and as quantiles (1–4). We further calculated deattenuated correlation coefficients between the PDS computed from the FFQs and 7DDR, correcting for within-person error in the 7DDR, using the following formula:

$$Pc=Po\sqrt{\left(1+\frac{\gamma }{k}\right)}$$

where *P_c_* is the corrected correlation coefficient between the FFQ and 7DDR, *Po* is the observed energy-adjusted correlation coefficient between the FFQ and 7DDR, γ is the ratio of within-to-between-person variation in the 7DDR PDS, and *k* is the number of diet record days recorded (*k* = 7 in this instance).

We also calculated deattenuated Pearson or Spearman correlation coefficients between the single components (as quantiles and absolute amounts) of the PDS from the FFQ and from the 7DDR, as well as energy (kcal) and macronutrients (carbohydrate, protein, fat). The single components (absolute amounts) of the PDS were log transformed after adding (0.0001 servings/day) to the data to allow the zeros to be fixed to a non-zero value as the components were positively skewed. We additionally analysed the absolute agreement between the two methods (FFQ and 7DDR) with the Bland–Altman method, which determines the average agreement between two methods by calculating the mean of their differences (FFQ-7DDR) against the mean intake of the two measures (FFQ + 7DDR/2). The 95% limits of agreement (LOA) provide an interval within which 95% of these differences are expected to fall.

Lastly, we calculated the proportion of participants correctly categorized (same quantile) and grossly misclassified (opposite quantile) for the PDS and single components. Statistical tests were 2-sided and *p* < 0.05 was considered statistically significant. The statistical analyses were conducted with Stata statistical software (Stata Statistical Software: Release 15. College Station, TX, USA).

## 3. Results

### 3.1. Development of the PDS

Table 1 reports the specific FFQ food items and their respective serving sizes that contributed to the PDS. Mean intake among the highest quintiles for each PDS component failed to meet the recommendations from the Portfolio Diet RCTs, as expected, with the exception of low dietary cholesterol intake (<200 mg/day) (Table S1).

Interitem correlations between the six individual score components were highest between phytosterols and plant protein, viscous fiber, nuts and MUFAs, with correlations ranging between 0.30 and 0.41; however, these correlations still suggested a sufficiently unique variance with the other components [54] (Table S2). In our sensitivity analysis assessing median vs. quintile cut-offs, the quintile approach resulted in more favorable results with LDL-C than when the median was used, which did not show significant inverse associations with LDL-C (data not shown), as expected. Finally, in the sensitivity analyses providing double weight to the low saturated fat/cholesterol component, no change was observed in the association with LDL-C (data not shown); thus, the PDS with equal weights for each component was used in the final analyses.

### 3.2. Validation of PDS by FFQ

Assessment of the PDS and its validity was based on quintiles of intake and with equal weight given to each component, as described above. The participants included in the concurrent validation subsample (*n* = 50) were different from those in the larger predictive validation group (*n* = 652) in that more were male and had a lower baseline BMI (Table 2). Mean intake by baseline FFQ was significantly higher for phytosterols in the predictive validation group, although no other differences were observed in overall PDS or the other five components.

Table 3 reports the mean total servings/day (or mg/day) for each of the quintiles of the six components at baseline and six months within the predictive validation sample. At each time period, mean intakes increased at least four-fold across quintiles, and were highest for viscous fiber compared to other PDS components (3.24 and 4.77 servings/day at highest quintile). In contrast, plant protein, nuts and MUFA intakes were low (less than 1 serving/day) in all but the highest quintile where mean intakes ranged from 1–2 servings/day. At six months compared to baseline, greater servings/day of viscous fiber and plant protein (although small for plant protein) were observed, as well as phytosterols (mg/day). Fewer servings/day of saturated fat/cholesterol sources at six months were also observed, with little change in nut and MUFA intake.

Table 4 shows the predictive validity results. Change in the PDS was inversely associated with change in LDL-C over six months, with a 1-point increase in score associated with a −0.01 mmol/L (95% confidence intervals (CI): −0.02, −0.002; *p* = 0.02) reduction in LDL-C. The odds of achieving a 5% reduction in LDL-C was also 5% greater in those who had a 1-point change in the PDS over six months (95% CIs: 1.01–1.10; *p* = 0.03). The Pearson correlation coefficient between the change in the PDS and change in LDL-C was −0.08 (*p* = 0.03).

Table 5, Table 6 and Table 7 show the concurrent validity results. The median intake was higher on the FFQ for all PDS components except phytosterols and the saturated fat/cholesterol sources, which were higher in the 7DDR (Table 5). The median intake for total energy, carbohydrates, protein and fat was higher on the 7DDR than the FFQ (Table 6). The Pearson correlation coefficients for the overall PDS estimated from the FFQ were reasonably correlated with those from the 7DDR, and ranged from 0.66 to 0.69 for the crude, energy-adjusted and deattenuated coefficients (Table 5). 

The energy-adjusted and deattenuated correlation coefficients ranged from 0.26 to 0.64 for the individual six components, with plant protein (servings/day) having the weakest correlation and phytosterols (mg/day) having the strongest correlation. Table 6 shows the Pearson correlation coefficients for energy intake and macronutrients (total fat, protein and carbohydrates). The energy-adjusted and deattenuated correlation coefficients ranged from 0.34 to 0.64, with energy intake (kcal/day) showing the weakest correlation and carbohydrates (g/day) showing the strongest correlation.

The PDS was then split into quartiles to further evaluate correlations and the ranking of participants by the FFQ and 7DDR (Table 7). The energy-adjusted and deattenuated Spearman correlation coefficients for quartiles of the PDS was 0.67, similar to the absolute score correlation. In addition, 48% of individuals were ranked into the same quartile of the PDS on both diet assessment tools. The percentage of individuals in opposite quartiles on the FFQ and 7DDR was 0%. The energy-adjusted and deattenuated Spearman correlation coefficients for quintiles of intake of the six components ranged from 0.36 to 0.54, with the saturated fat/cholesterol and plant protein components having the weakest correlations, and phytosterols, MUFAs and nuts having the strongest correlations. On average, 32% of individuals were grouped into the same quintile on both diet tools (ranging from 24% for phytosterols and 38% for plant protein). Gross misclassification (percentage of individuals grouped in opposite quintiles) ranged from 0–4% for all components.

**Figure 2:**
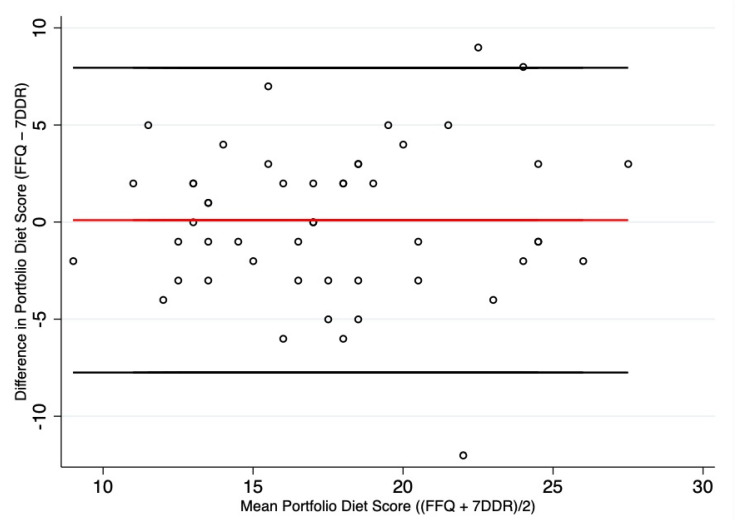
Mean differences of the PDS derived from the FFQ and 7DDR. Abbreviations: FFQ, food frequency questionnaire; 7DDR, 7-day diet record. Solid black lines are the upper and lower 95% LOA (−7.75 to 7.94). Red line is the mean difference (0.10).

Figure 2 and Figures S1–S10 show the Bland–Altman plots to assess mean agreement between the FFQ and 7DDR estimates for absolute intake of the PDS, its six PDS components, energy, total fat, protein and carbohydrate. In general, the Bland–Altman plots showed good agreement for the PDS, all PDS components and macronutrients assessed. Although the mean difference between the two dietary methods was close to 0 for the PDS and its individual components, the FFQ appeared to slightly overestimate 4 of the 6 components (plant protein, viscous fiber, nuts, MUFAs) and underestimate phytosterols and saturated fat, compared to the 7DDR (Figures S6–S10). There also appeared to be better agreement at lower intake than higher intake as evidenced by a clustering of data at low intake and a greater scatter at high intake for the plant protein, nuts, viscous fiber and MUFAs Bland–Altman plots; however, this finding needs to be interpreted with caution as low consumption of these foods in both the FFQ and 7DDR cannot have a large difference between the two diet assessment methods (Figures S6–S9).

## 4. Discussion

The current study developed a PDS and assessed its predictive (*n* = 652) and concurrent validity (*n* = 50) by comparing intake estimates from an FFQ to LDL-C and a 7DDR among overweight adults in Toronto. We developed a mainly food-based PDS based on population-specific intake using quintile cut-offs and with equal weight for each component of the score. We observed that the change in the PDS was inversely associated with a reduction in LDL-C and with the odds of achieving a 5% reduction in LDL-C over six months. Compared with data from a 7DDR, the FFQ showed reasonable concurrent validity and agreement to rank participants on the overall PDS, with a moderately high energy-adjusted and deattenuated correlation coefficient of 0.69.

There was also acceptable validity and agreement between the 7DDR and FFQ for most absolute intakes of individual components of the PDS, energy and macronutrients; however, correlation coefficients were poor (below 0.3) for plant protein and MUFAs and only slightly better for nuts (r = 0.33) [55]. There was also a small overestimation of several Portfolio Diet components by the FFQ compared to the 7DDR, particularly at higher intake. The lower correlation coefficients for plant protein, MUFAs, and nuts suggest that intake of these components may be more difficult to estimate on the FFQ than with a 7DDR compared to the other components. Alternatively, it is possible that these components were better assessed by the FFQ as they may be more episodically consumed (i.e., not daily) than the other components, and the 7DDR may not have captured the specific days when these components were consumed [56]. Additionally, quintiles of intake resulted in correlation coefficients that were >0.1 higher than those based on absolute intake but only for these same components (plant protein, nuts and MUFAs). Therefore, these potentially episodically consumed components may be better assessed by ranking since mean consumption in all but the highest quintile was low (less than 1 serving/day), there was better agreement at low intakes between the FFQ and the 7DDR, and any discordant assessment between the methods at higher intakes may not have substantially reduced the ability to rank participants’ consumption of these components.

During the development phase of our PDS, we observed that basing the score cut-offs on quintiles rather than medians resulted in significant inverse associations with LDL-C. This finding is in line with our hypothesis and previous literature that has shown that loss of information is reduced with four or five groups and diagnostic accuracy is improved by providing better discriminating power to detect differences in health outcomes [35,57,58]. We also observed that providing more weight to the low saturated fat/cholesterol component did not change the association with LDL-C. Many other diet scores have not reported or examined weighting of their score components, and most provide equal weight to each score component [24,31,34].

To the best of our knowledge, this is the first PDS created in the literature; therefore, a direct comparison of our score with other validation studies is not possible. Studies evaluating the predictive validity of other dietary patterns with blood lipids such as cholesterol do exist, however, but are limited. The prudent [59], Healthy Eating Index (HEI) [60,61], Diet Quality Index Revised (DQI-R) [62], and the plant-based diet indices [63] have been evaluated for associations with total cholesterol (TC) and/or LDL-C; however, most studies reported TC only. The prudent dietary pattern determined by principal component analysis and intake data from a 131-item Willet FFQ used with 127 men from the Health Professionals Follow-up Study (HPFS) was reported to have Pearson correlation coefficients with TC ranging from −0.08 to −0.12, which is similar to our LDL-C correlation coefficient of −0.08 [59]. Furthermore, DQI-R scores using the same 131-item Willet FFQ and in the HPFS cohort, were inversely correlated with TC (r = −0.22) [62]. In contrast, no significant correlations between HEI scores determined from a three-day diet record and TC concentrations were found in a validation study of 340 women from the University of Michigan Hospital’s Breast Care Center [61]. Likewise, no significant correlations were found between the plant-based diet indices determined from a 152-item Willett FFQ and TC in 720 women and 634 men from the Women’s and Men’s Lifestyle Validation Studies of the HPFS and Nurses Health Studies I and II [63]. Correlation coefficients were assessed between the HEI score from a 24-h dietary recall and LDL-C levels in a subsequent validation study in 6979 men and women from the National Health and Nutrition Examination Survey (NHANES) III, and small but also significant correlation coefficients (r = −0.04) were seen [60]. This NHANES validation study only collected one day of intake from participants; therefore, intake may have been underestimated [64]. Our study saw significant inverse correlations between change in the PDS and change in LDL-C, which is the main target of the Portfolio Diet, and may also explain why we saw significant relationships with this CVD risk factor and some of the other validation studies did not.

For the concurrent validation, data on dietary patterns is also scarce. As discussed previously, the prudent and Western dietary patterns derived from principal component analysis in the HPFS cohort had energy-adjusted and deattenuated correlation coefficients between the FFQ and two 1-week diet records that ranged from 0.45 to 0.74 [59]. Similar deattenuated correlation coefficients (0.41 to 0.73) between a 60-item FFQ and four 1-week diet records were also found for the prudent, Western and other dietary patterns derived from principal component analysis in 111 women from the Swedish Mammography cohort [65]. Our finding of an energy-adjusted and deattenuated correlation coefficient (r = 0.69) for the PDS is within the range reported in these two studies. Of more direct relevance to our PDS, a validation study of the plant-based diet indices found energy-adjusted and deattenuated correlation coefficients between the indices from a 152-item Willet FFQ and two 1-week diet records ranging from 0.62 to 0.67, which are also similar to our findings [63]. A further study assessed the validity of two modified Mediterranean diet scores, based on tertile distribution of food consumption, in 107 men and women from the REGICOR study in Spain [66]. The energy-adjusted and deattenuated correlation coefficients between a 166-item FFQ and ten 24-h recalls for both Mediterranean diet scores ranged from 0.48 to 0.61, which is slightly weaker, but similar, to our findings. In addition, they found reasonable agreement and gross misclassification through their Bland–Altman and opposite quantile analysis, also in line with our results [66]. Both their modified Mediterranean diet score (score range 10–30) and the Mediterranean-like diet score (score range 13–39) had limits of agreement (LOA) that ranged from −7 to +7 in Bland–Altman plots comparing mean intakes from the FFQ and multiple 24-h recalls. Additionally, our LOAs were well within the boundaries of 50%−200%, identified by these and other authors as being reasonable [66,67]. Overall, our study and other literature are in agreement that dietary patterns assessed through the use of FFQs provide reasonably valid measures of intake and ranking compared to multiple days of diet records or 24-h recalls.

Energy-adjusted and deattenuated correlation coefficients for the six individual Portfolio Diet components ranged from 0.26–0.64 for absolute intakes (servings/day or mg/day) and 0.36–0.54 for quintile rankings (1–5) in our study, which is comparable to the results of absolute intakes in other studies where similar groupings to our PDS were available [45,66,68]. Of particular interest is the phytosterol component, as this was a new nutrient added to the FFQ and 7DDR data in our study. Phytosterols showed correlation coefficients of 0.54 for rank in quintiles and a correlation coefficient of 0.64 for absolute intake, both indicating reasonable validity. These results are consistent with a previous study that estimated the phytosterol intake from a 178-item FFQ against twelve 24-h recalls in the EPIC-Netherlands cohort. The researchers reported an energy-adjusted correlation coefficient of 0.59 between the two methods [45]. Further research assessed the energy-adjusted and deattenuated correlation coefficients and agreement between the individual components of a Mediterranean diet score [66]. Food categories similar to ours included nuts, legumes and olive oil, where correlation coefficients of absolute intakes obtained through a 166-item FFQ and 24-h recalls were 0.36, 0.25 and 0.33, respectively [66]. These results are in line with the correlation coefficients we found with absolute intakes of nuts (0.33), plant protein (0.26) and MUFAs (0.28). For the misclassification analysis in the Mediterranean diet score study, more participants were in either the same or opposite tertiles to our study [66]. This discrepancy may be related to analyzing tertiles instead of quintiles of intake, as quintiles provide a larger range of options to classify participants. Additionally, Feskanich et al. (1993) conducted a foods validation study using a 131-item Willet FFQ in 127 men from the HPFS where observed energy-adjusted and deattenuated correlations between two 1-week diet records and the FFQs were 0.72 for peanut butter, 0.17 for other nuts, 0.34 for beans/lentils and 0.56 for tofu/soybeans [68]. These correlations are generally stronger than those for similar food components in our study; however, our nut component (r = 0.33) included both peanut butter and other nuts, and our plant protein component (r = 0.26) combined beans/lentils and tofu/soy foods and are therefore not directly comparable. Nonetheless, the stronger correlation coefficients observed in the previous study may be explained by the larger sample size and greater number of diet records in their study compared to ours.

Strengths of our study include the various assessments conducted when developing the score, as well as the use of multiple methods to assess the validity and agreement between the two diet assessment tools. We assessed the scores ability to predict changes in LDL-C and correlation with LDL-C, the main target of the Portfolio Diet. Using change in LDL-C, rather than baseline LDL-C as assessed in other validation studies, is also a strength as variability due to individual genetic or other determinants is removed [69]. We also assessed concurrent validity of the PDS using correlation coefficients, Bland–Altman plots and cross-classification. All methods showed reasonable validity of the FFQ to assess the PDS, compared to a 7DDR, in overweight adults. Another strength of our study is the addition of relevant foods to the FFQ to better capture adherence to the Portfolio Diet. Additional questions on other high MUFA sources would have further strengthened our ability to assess adherence to this component of the diet. For example, we were not able to quantify total olive oil intake and questions on other high MUFA oils, such as canola or sunflower, were not included on the FFQ.

Nevertheless, there are limitations to the present study. We used a 7DDR as the reference method, which is also a self-reported diet assessment method; therefore, it is not error free and errors between the FFQ and 7DDR may be correlated. Ideally, the 7DDR would have been weighed diet records with more than seven days of recording. On the other hand, the FFQ asked about usual intake over the past month. Therefore, seven days may be reasonable for this time frame. However, non-consecutive days would have better captured within-person variation [69] and may have resulted in stronger correlations for potential episodically consumed foods such as plant protein. It is also important to note that our sample size (n = 50) for the concurrent validation analysis is small, and preferably would have been larger and had adequate numbers of males and females [69]. We were unable to examine sex differences due to the small sample size and because our population was mostly female (76% for predictive and 62% for concurrent validation analyses). The population also included overweight adults and therefore the results may not be applicable to the general population. Lastly, the correlation between increasing PDS with lower LDL-C was not overly strong, and we would expect stronger correlations if intake of the individual Portfolio Diet components were higher in this population. The reductions in LDL-C were likely driven by increased intake of the more commonly consumed foods containing viscous fiber and phytosterols, and decreased intake of saturated fat/cholesterol sources over the six months. In general, there was limited consumption of plant protein, nuts and MUFAs in the current study, except among those in the highest quintiles of intake, which is in agreement with more recent data from the 2015 Canadian Community Health Survey, where nuts, seeds and legumes only contributed 5% of total protein intake [70]. Greater intake of the Portfolio Diet components would likely have resulted in stronger inverse correlations with LDL-C.

Future studies that use our PDS, which is based on population-specific cut-offs, will need to consider that intake of the six individual components and results may vary largely among populations. The inclusion of more ethnically diverse populations or vegetarians may also improve the examination of PDS food components that were minimally consumed in this study (e.g., plant proteins, nuts). Comparison of dietary intake across populations will help determine the level of Portfolio Diet adherence that is associated with a lower risk of disease outcomes. Lastly, whether the PDS can predict disease incidence in diverse populations will be a critical assessment of its validity. To date, we have assessed our PDS in an overweight/obese population with metabolic syndrome in the PREDIMED-Plus clinical trial cohort and with incident CVD outcomes in postmenopausal women in the Women’s Health Initiative (WHI). Greater adherence to our PDS was significantly associated with lower HbA1c, fasting plasma glucose, triglycerides, BMI and waist circumference over one year in PREDIMED-Plus [17], and was significantly associated with a lower risk of CVD, coronary heart disease and heart failure in the WHI [18]. These two studies provide further evidence that the PDS can predict improvements in several cardiometabolic risk factors and diseases.

## 5. Conclusions

The PDS predicts lower LDL-C and shows reasonable concurrent validity over six months in overweight adults. Future studies are needed to examine diet–disease relationships in prospective cohort studies and whether the PDS can further reliably predict clinical disease outcomes, such as lower CVD risk.

## Figures and Tables

**Table 1:** Portfolio Diet components and food items included in the FFQ.

Portfolio Diet Components	Food Items (Serving Sizes) in FFQ ^a^
Plant protein, servings/day	Tofu, soybeans, or vegetable protein (3–4 oz); peas or lima beans (1/2 cup); lentils or beans, kidney, pinto, black-eyed, chickpeas, etc. (1/2 cup); soymilk (8 oz glass)
Viscous fiber, servings/day	Cold breakfast cereal, Bran buds (1 cup); cooked oatmeal (1 cup); cooked oat bran (1 cup); oat bran added to food (1 tbsp); barley (1 cup); fresh apples or pears (1); applesauce (1/2 cup); oranges, tangerines, clementines (1); grapefruit (1/2); strawberries, raspberries or blackberries (1/2 cup); blueberries (1/2 cup); okra (1/2 cup); eggplant (1/2 cup); Metamucil supplement (2 tsp) ^b^
Nuts, servings/day	Peanut butter (1 tbsp); almond butter (1 tbsp); peanuts (1 oz); almonds (1 oz); walnuts (1 oz); other nuts (1 oz)
Phytosterols, mg/day	Estimated from all plant foods
MUFAs ^c^, servings/day	Avocado (1/2); olive oil, added to food or bread (1 tbsp); olive oil salad dressing (2 tbsp)
Saturated fat/cholesterol, servings/day	Whole milk (8 oz); cream (1 tbsp); ice cream (1/2 cup); other cheese (1 oz); cream cheese (1 oz); eggs with yolk (1); bacon (2 slices); poultry with skin (5 oz); beef/pork hot dogs (1); chicken/turkey hot dogs (1); salami, bologna or other processed meat (2 oz); lean hamburger (1 patty); regular hamburger (1 patty); mixed meals with red meat (1); pork (5 oz); beef/lamb (5 oz); beef liver (4 oz); chicken liver (1 oz); other organ meats (3 oz); butter (1 tsp); Salami, bologna or other processed meat sandwiches (1)

Semi-colons between food items indicate separate line items on the FFQ. Abbreviations: FFQ, food frequency questionnaire; MUFAs, monounsaturated fatty acids. ^a^ Six items relevant to the Portfolio Diet were added to the FFQ (cooked oat bran, barley, eggplant, almond butter, almonds, and walnuts); FFQ changes were made to expand or clarify inclusions (e.g., raspberries and blackberries were added to the item for strawberries). Coding rules for open-ended questions about the usual type and brand of cold breakfast cereals (e.g., Bran Buds), cooking oils, margarine, and multivitamin supplements were revised for the Canadian market. ^b^ Serving size of 2 tsp was assigned based on recommendations in Metamucil instructions. Intake frequency of once per week was assigned as FFQ asked about supplements taken “at least once per week”. ^c^ Total olive oil not captured, as frying/sautéing and baking questions did not quantify olive oil consumption.

**Table 2:** Baseline characteristics of the participants included in the predictive and concurrent validation analyses.

Characteristic, Mean (SD)/*n* (%)	Predictive Validation (*n* = 652)	Concurrent Validation (*n* = 50)	*p*-Value
Age, year	45.2 (12.6)	45.3 (11.5)	0.96
Sex, female	493 (76)	31 (62)	0.03
Current smoker	45 (7)	3 (7)	0.07
Exercise level			
Low	104 (16)	7 (14)	0.62
Medium	462 (71)	34 (68)	
High	86 (13)	9 (18)	
Cholesterol-lowering medication use	43 (7)	2 (4)	0.47
Blood pressure lowering medication use	64 (10)	6 (12)	0.62
BMI	32.1 (5.42)	28.9 (4.57)	<0.001
Family history of CVD	288 (44)	20 (40)	0.58
Total energy intake, kcal/d	1995 (707)	2056 (822)	0.56
Baseline LDL-C, mmol/L	3.24 (0.80)	3.25 (0.70)	0.93
Highest education completed			0.54
High school or less	212 (33)	14 (28)	
Undergraduate/college degree	294 (45)	25 (50)	
Graduate degree	144 (22)	11 (22)	
Ethnicity ^a^			<0.001
Caucasian	416 (64)	25 (50)	
Asian	70 (11)	3 (6)	
African & Caribbean	49 (8)	0 (0)	
Other	58 (9)	1 (2)	
Unknown	59 (9)	21 (42)	
Control group	270 (41)	27 (54)	0.08
PDS ^b^, mean (SD), min–max	18.1 (4.31) 7–30	17.6 (4.86) 8–29	0.43
Plant protein, servings/day ^b^	0.41 (0.67)	0.46 (0.56)	0.61
Viscous fiber, servings/day ^b^	1.38 (1.17)	1.28 (1.35)	0.56
Nuts, servings/day ^b^	0.67 (0.86)	0.90 (1.28)	0.08
Phytosterols, mg/day ^b^	337 (158)	284 (171)	0.02
MUFAs, servings/day ^b^	0.65 (0.84)	0.55 (0.64)	0.41
Saturated fat/cholesterol, servings/day ^b^	2.48 (1.82)	2.89 (2.26)	0.13

Data are shown as mean (SD) or n (%) unless otherwise stated. Abbreviations: BMI, body mass index; CVD, cardiovascular disease; LDL-C, low-density lipoprotein cholesterol; MUFAs, monounsaturated fatty acids; PDS, Portfolio Diet score; y, year. ^a^ More participants in the concurrent validation subsample had an unknown ethnicity as this information was not collected at the beginning of the study when the 7DDRs were completed; therefore, true differences may not exist. ^b^ Dietary variables were assessed by baseline FFQ; intake of PDS and components based on specific food items listed in Table 1.

**Table 3:** Mean daily intake of the Portfolio Diet components by quintile and assigned scores, assessed by FFQ at baseline and 6 months (*n* = 652).

	Baseline					6 Months				
Component	Q1 (1 Point)	Q2 (2 Points)	Q3 (3 Points)	Q4 (4 Points)	Q5 (5 Points)	Q1 (1 Point)	Q2 (2 Points)	Q3 (3 Points)	Q4 (4 Points)	Q5 (5 Points)
Plant protein, servings/day	0.03	0.11	0.19	0.43	1.31	0.05	0.15	0.37	0.75	1.59
Viscous fiber, servings/day	0.25	0.66	1.08	1.59	3.24	0.51	1.17	1.74	2.60	4.77
Nuts, servings/day	0.03	0.14	0.38	0.81	1.96	0.05	0.17	0.39	0.72	1.94
Phytosterols, mg/day	164	240	310	389	587	182	274	354	443	690
MUFAs, servings/day	0.02	0.12	0.40	0.82	2.03	0.02	0.14	0.45	0.83	2.07
Saturated fat/cholesterol ^a^, servings/day	5.50	3.05	2.16	1.42	0.62	4.29	2.47	1.71	1.16	0.52

Abbreviations: FFQ, food frequency questionnaire; MUFAs, monounsaturated fatty acids; Q, quintile. All components reported as servings/day except for phytosterols (mg/day). Intake of PDS components based on specific food items listed in Table 1. ^a^ Higher quintiles represent higher intake; however, high intake and high quintiles of saturated fat/cholesterol received lower scores.

**Table 4:** Change in PDS and concomitant change in LDL-C from baseline to 6 months (*n* = 652).

LDL-C (mmol/L)	Per 1-Unit Increase (β Coefficients (95%CI))	*p*-Value	5% Reduction in LDL-C (OR (95% CI))	*p*-Value	Pearson Correlation Coefficient	*p*-Value
Model 1 *	−0.01 (−0.02, −0.002)	0.02	1.05 (1.01–1.10)	0.01	−0.08	0.03

* Model 1 adjusted for sex, age, baseline LDL-C, energy intake, cholesterol-lowering medication use, ethnicity, BMI, smoking status, family history of CVD, education, physical activity, and intervention group. Intake of PDS based on specific food items listed in Table 1.

**Table 5:** Daily absolute intake of the individual Portfolio Diet components and Pearson correlation coefficients between estimates by the baseline FFQ and 7DDR (*n* = 50).

	FFQ	7DDR	Pearson Correlation Coefficients		
	Median (Min–Max)	Median (Min–Max)	Unadjusted, r (95% CI)	Energy-Adjusted, r (95% CI)	Energy-Adjusted and Deattenuated, r (95% CI)
PDS, points	17.6 (8–29)	17.0 (9–28)	0.66 (0.47, 0.80)	0.65 (0.45, 0.79)	0.69 (0.48, 0.85)
Plant protein, servings/day	0.19 (0.00–2.85)	0.17 (0.00–2.77)	0.25 (−0.03, 0.49)	0.25 (−0.04, 0.49)	0.26 (−0.04, 0.52)
Viscous fiber, servings/day	0.88 (0.05–6.93)	0.53 (0.00–2.23)	0.42 (0.15, 0.62)	0.40 (0.14, 0.61)	0.44 (0.15, 0.67)
Nuts, servings/day	0.50 (0.00–6.72)	0.37 (0.00–5.36)	0.40 (0.14, 0.61)	0.31 (0.04, 0.54)	0.33 (0.04, 0.58)
Phytosterols, mg/day	234 (68–964)	316 (108–780)	0.40 (0.13, 0.61)	0.59 (0.38, 0.75)	0.64 (0.41, 0.80)
MUFAs, servings/day	0.44 (0.00–3.29)	0.29 (0.00–2.10)	0.30 (0.02, 0.53)	0.26 (−0.02, 0.50)	0.28 (−0.02, 0.55)
Saturated fat/cholesterol, servings/day	2.25 (0.08–10.9)	3.37 (0.08–7.44)	0.37 (0.10, 0.59)	0.37 (0.10, 0.59)	0.41 (0.11, 0.65)

Abbreviations: CI, confidence intervals; FFQ, food frequency questionnaire; MUFAs, monounsaturated fatty acids; PDS, Portfolio Diet score; 7DDR, 7-day diet record. Intake of PDS and components based on specific food items listed in Table 1. The individual PDS components were log-transformed.

**Table 6:** Daily absolute intake of the calories and macronutrients and Pearson correlation coefficients between estimates by the baseline FFQ and 7DDR (*n* = 50).

	FFQ	7DDR	Pearson Correlation Coefficients		
	Median (Min–Max)	Median (Min–Max)	Unadjusted, *r* (95% CI)	Energy-Adjusted, *r* (95% CI)	Energy-Adjusted and Deattenuated, *r* (95% CI)
Energy, kcal/day	2049 (744–4690)	2114 (1171–3729)	0.31 (0.04, 0.54)	--	0.34 (0.04, 0.58)
Carbohydrate, g/day	233 (80–656)	270 (100–417)	0.31 (0.03, 0.54)	0.59 (0.37, 0.75)	0.64 (0.40, 0.81)
Total protein, g/day	83 (26–178)	94 (54–180)	0.23 (−0.05, 0.48)	0.38 (0.11, 0.59)	0.41 (0.12, 0.65)
Total fat, g/day	70 (18–187)	79 (37–143)	0.42 (0.15, 0.62)	0.53 (0.29, 0.70)	0.57 (0.31, 0.76)

Abbreviations: CI, confidence intervals; FFQ, food frequency questionnaire; 7DDR, 7-day diet record.

**Table 7:** Correlation coefficients and agreement in the quantiles of the PDS and individual Portfolio Diet components between the baseline FFQ and 7DDR (*n* = 50).

	Spearman Correlation Coefficients				
	Unadjusted, *r* (95% CI)	Energy-Adjusted, *r* (95% CI)	Energy-Adjusted and Deattenuated, *r* (95% CI)	Same Quantile ^a^, *n* (%)	Opposite Quantile ^b^, *n* (%)
PDS	0.69 (0.51, 0.81)	0.65 (0.45, 0.79)	0.67 (0.47, 0.83)	24 (48)	0 (0)
Plant protein	0.35 (0.07, 0.57)	0.36 (0.09, 0.58)	0.38 (0.10, 0.62)	19 (38)	1 (2)
Viscous fiber	0.44 (0.18, 0.64)	0.39 (0.13, 0.60)	0.42 (0.14, 0.64)	18 (36)	2 (4)
Nuts	0.58 (0.36, 0.74)	0.49 (0.25, 0.68)	0.53 (0.27, 0.73)	17 (34)	0 (0)
Phytosterols	0.43 (0.18, 0.64)	0.51 (0.27, 0.69)	0.54 (0.29, 0.73)	12 (24)	0 (0)
MUFAs	0.57 (0.35, 0.73)	0.50 (0.25, 0.68)	0.54 (0.27, 0.74)	18 (36)	1 (2)
Saturated fat/cholesterol	0.31 (0.04, 0.54)	0.34 (0.07, 0.57)	0.36 (0.07, 0.71)	13 (26)	0 (0)

Abbreviations: CI, confidence intervals; FFQ, food frequency questionnaire; MUFAs, monounsaturated fatty acids; PDS, Portfolio Diet score; 7DDR, 7-day diet record. Intake of PDS and components based on specific food items listed in Table 1. ^a^ Same quantile is between Q1 to Q4 for the PDS and Q1 and Q5 for the individual PDS components. ^b^ Opposite quantile is Q1 to Q4 for the PDS and Q1 to Q5 for the individual PDS components.

## Data Availability

Data is contained within the article.

## Supplementary Materials

The following are available online at https://www.mdpi.com/article/10.3390/nu13082850/s1, Table S1: Estimated mean ABSOLUTE intakes for the PDS components for Q1 through Q5 from the FFQ at baseline and 6 months compared to the Portfolio Diet trial recommendations (*n* = 652), Table S2: Inter-item correlations between the individual Portfolio Diet components contributing to the PDS (*n* = 652), Figure S1: Mean differences of the total energy intake (kcal/day) derived from the FFQ and 7DDR (*n* = 50), Figure S2: Mean differences of the carbohydrate intake (g/day) derived from the FFQ and 7DDR (*n* = 50), Figure S3: Mean differences of total protein intake (g/day) derived from the FFQ and 7DDR (*n* = 50), Figure S4: Mean differences of the total fat intake (g/day) derived from the FFQ and 7DDR (*n* = 50), Figure S5: Mean differences of plant protein (servings/day) derived from the FFQ and 7DDR (*n* = 50), Figure S6: Mean differences of viscous fiber (servings/day) derived from the FFQ and 7DDR (*n* = 50), Figure S7: Mean differences of nuts (servings/day) derived from the FFQ and 7DDR (*n* = 50), Figure S8: Mean differences of phytosterols (mg/day) derived from the FFQ and 7DDR (*n* = 50), Figure S9: Mean differences of MUFAs (servings/day) derived from the FFQ and 7DDR (*n* = 50), Figure S10: Mean differences of Saturated Fat/Cholesterol (servings/day) derived from the FFQ and 7DDR (*n* = 50).
