# Supplementary material for: Development of a Portfolio Diet Score and Its Concurrent and Predictive Validity Assessed by a Food Frequency Questionnaire
Source: Nutrients. 2021 Aug 19;13(8):2850. doi: 10.3390/nu13082850 (PMC8398786; doi:10.3390/nu13082850)
Supplement: Supplementary file 1 [file nutrients-13-02850-s001.zip › nutrients-1283073-supplementary.pdf]

Supplemental Material:

Table S1: Estimated mean absolute intakes for the PDS components for Q1 through Q5 from the FFQ at baseline and 6 months compared to the Portfolio diet trial recommendations (n=652)

Table S2: Inter-item correlations between the individual Portfolio Diet components contributing to the PDS (n=652)

Figure S1: Mean differences of the total energy intake (kcal/day) derived from the FFQ and 7DDR (n=50)

Figure S2: Mean differences of the carbohydrate intake (g/day) derived from the FFQ and 7DDR (n=50)

Figure S3: Mean differences of total protein intake (g/day) derived from the FFQ and 7DDR (n=50)

Figure S4: Mean differences of the total fat intake (g/day) derived from the FFQ and 7DDR (n=50)

Figure S5: Mean differences of plant protein (servings/day) derived from the FFQ and 7DDR (n=50)

Figure S6: Mean differences of viscous fiber (servings/day) derived from the FFQ and 7DDR (n=50)

Figure S7: Mean differences of nuts (servings/day) derived from the FFQ and 7DDR (n=50)

Figure S8: Mean differences of phytosterols (mg/day) derived from the FFQ and 7DDR (n=50)

Figure S9: Mean differences of MUFAs (servings/day) derived from the FFQ and 7DDR (n=50)

Figure S10: Mean differences of Saturated Fat/Cholesterol (servings/day) derived from the FFQ and 7DDR (n=50)

This supplementary material has been provided by the authors to give readers additional information about their work.

Table S1: Estimated mean absolute intakes for the PDS components for Q1 through Q5 from the FFQ at baseline and 6 months compared to the Portfolio Diet trial recommendations (n=652)

	**Plant Protein** (g/day)	**Viscous Fiber** (g/day)	**Nuts** (g/day)	**Phytosterols** (mg/day)	**MUFAs** (g/day)	**Saturated fat** (g/day)	**Cholesterol** (mg/day)
BASELINE							
Q1	0.3	0.5	0.45	164	0.3	42.4	463.6
Q2	1.1	1.32	2.1	240	1.8	31.4	299.4
Q3	1.9	2.16	5.7	310	6	21.0	240.1
Q4	4.3	3.18	12.2	389	12.3	16.4	183.8
Q5	13.1	6.48	29	587	30.5	10.5	107.9
6 MONTHS							
Q1	0.5	1.02	0.75	182	0.3	36.3	436.4
Q2	1.5	2.34	2.55	274	2.1	24.0	285.1
Q3	3.7	3.48	5.9	354	6.75	18.8	225.3
Q4	7.5	5.2	10.8	443	12.5	14.4	171.3
Q5	15.9	9.54	29.1	690	31.1	9.3	97.3
Recommendations	50g daily	20g daily	45g daily	2g daily	45g daily	<7% of energy*	<200mg/day

Abbreviations: FFQ, food frequency questionnaire; MUFAs; monounsaturated fatty acids; PDS, Portfolio Diet score; Q=quintile

The absolute amounts for plant protein, viscous fiber, nuts and MUFAs were estimated by using the approximate gram amount contained in each food most commonly consumed in the category. Most of the plant protein came from lentils/beans, therefore total plant protein in grams was estimated based on this food (1/2 cup = ~10g protein). For viscous fiber, apples were the most commonly consumed food, therefore viscous fiber in grams was estimated based on apples (1 apple = ~2 g viscous fiber). For nuts, the most commonly

food consumed was peanut butter (1 tbsp = ~15g nuts). For MUFAs, olive oil was the most commonly consumed (1 tbsp = ~15g MUFA). These targets are based on data for determining adherence to the ongoing PortfolioEx randomized controlled trial (ClinicalTrials.gov Identifier: NCT02481466). Phytosterols were estimated by our group and reflect the data described in the main paper. The most commonly consumed foods in the saturated/cholesterol category were other cheese, eggs, and butter, however, total saturated fat (g/day) and cholesterol (mg/day) are available as a nutrient from the FFQ (unlike the other components), so the above table reflects this data.

*~15-16g of saturated fat is 7% of energy, based on mean energy intake of 1995 at baseline, and mean energy intake of 1944 kcal at 6 months

Intake of PDS and components based on specific food items listed in Table 1.

Table S2: Interitem correlations between the individual Portfolio Diet components contributing to the PDS (n=652)

Component	Plant protein	Viscous fiber	Nuts	Phytosterols	MUFAs	SFA/cholesterol
**Plant protein**	1.00	--	--	--	--	--
**Viscous fiber**	0.18	1.00	--	--	--	--
**Nuts**	-0.01	0.02	1.00	--	--	--
**Phytosterols**	0.34	0.41	0.37	1.00	--	--
**MUFAs**	-0.01	0.04	0.17	0.30	1.00	--
**SFA/cholesterol**	-0.09	0.05	0.06	0.11	0.05	1.00

Interitem correlations examine the extent in which scores on one item are related to scores on all other items.
Abbreviations: MUFAs; monounsaturated fatty acids; PDS, Portfolio Diet score; SFA, saturated fatty acids.

Intake of PDS and components based on specific food items listed in Table 1.

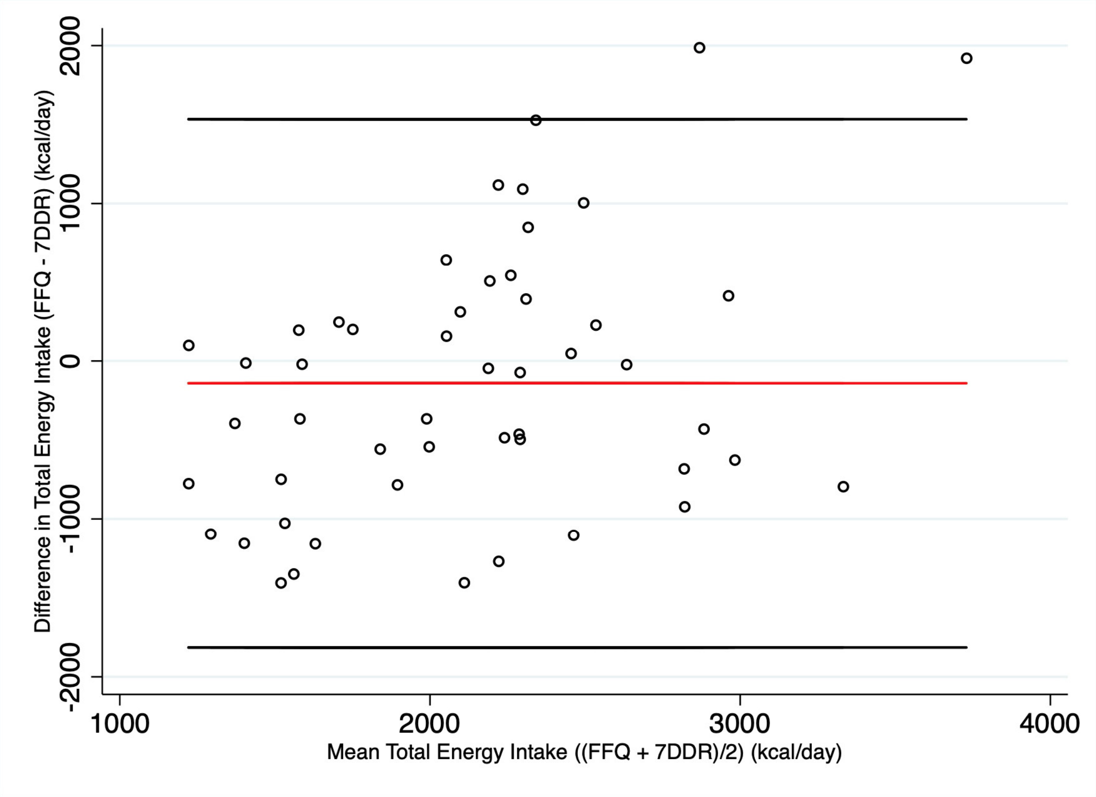

Figure S1: Mean differences of the total energy intake (kcal/day) derived from the FFQ and 7DDR (n=50). Solid black lines are the upper and lower 95% LOA (-1814 to 1533). Red line is the mean difference (-140.5).

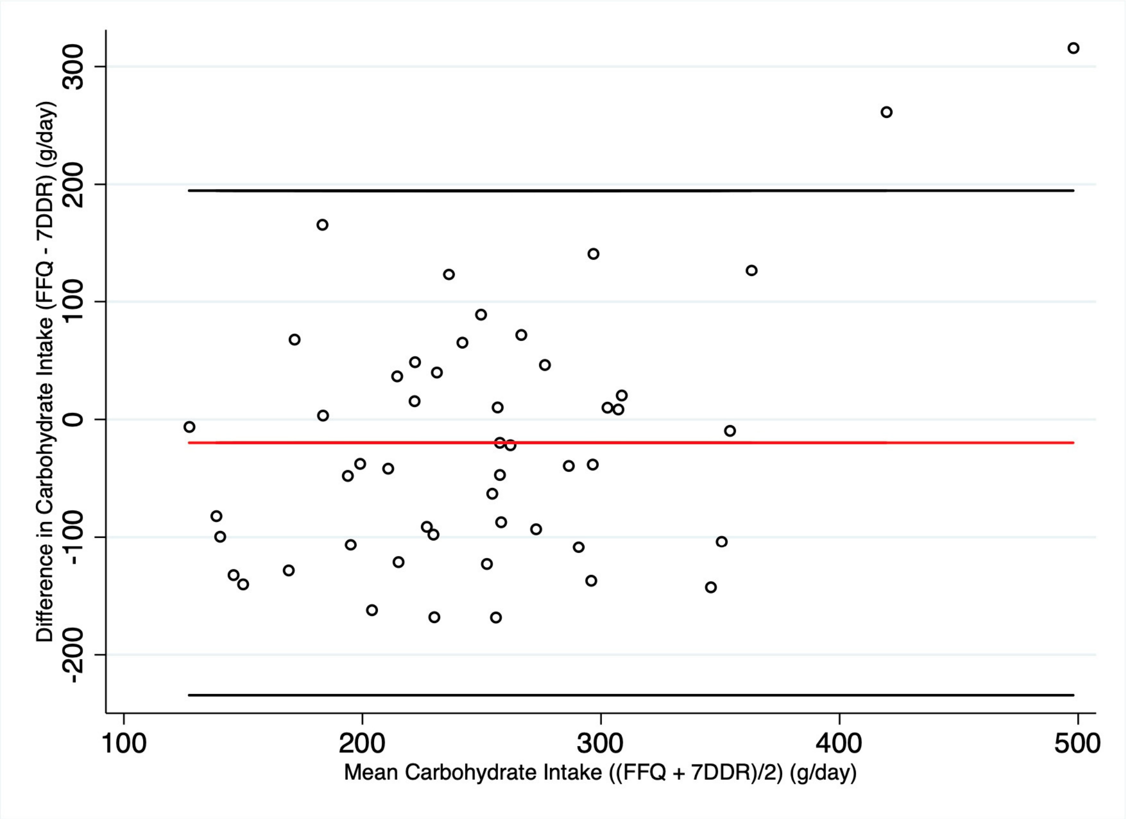

Figure S2: Mean differences of the carbohydrate intake (g/day) derived from the FFQ and 7DDR (n=50). Solid black lines are the upper and lower 95% LOA -234.2 to 194.6). Red line is the mean difference (-19.8).

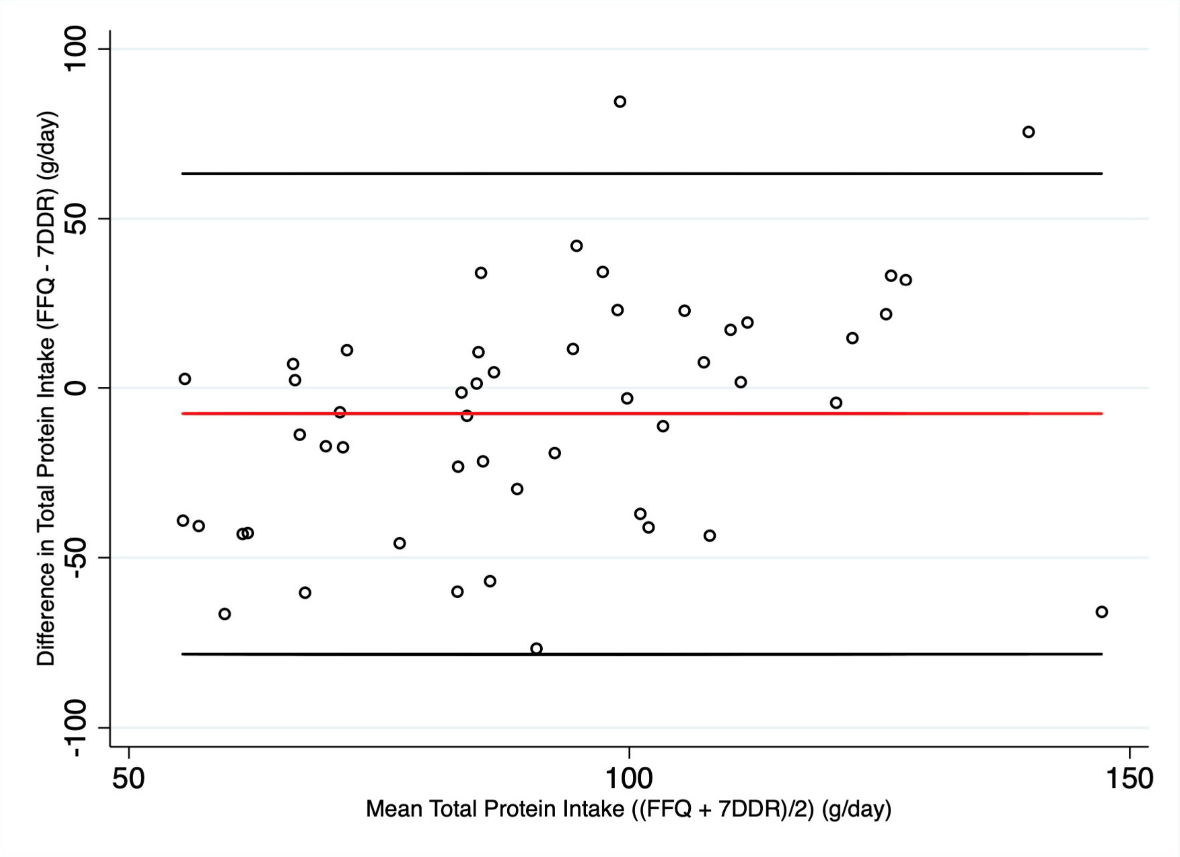

Figure S3: Mean differences of total protein intake (g/day) derived from the FFQ and 7DDR (n=50). Solid black lines are the upper and lower 95% LOA -78.4 to 63.3). Red line is the mean difference (-7.57).

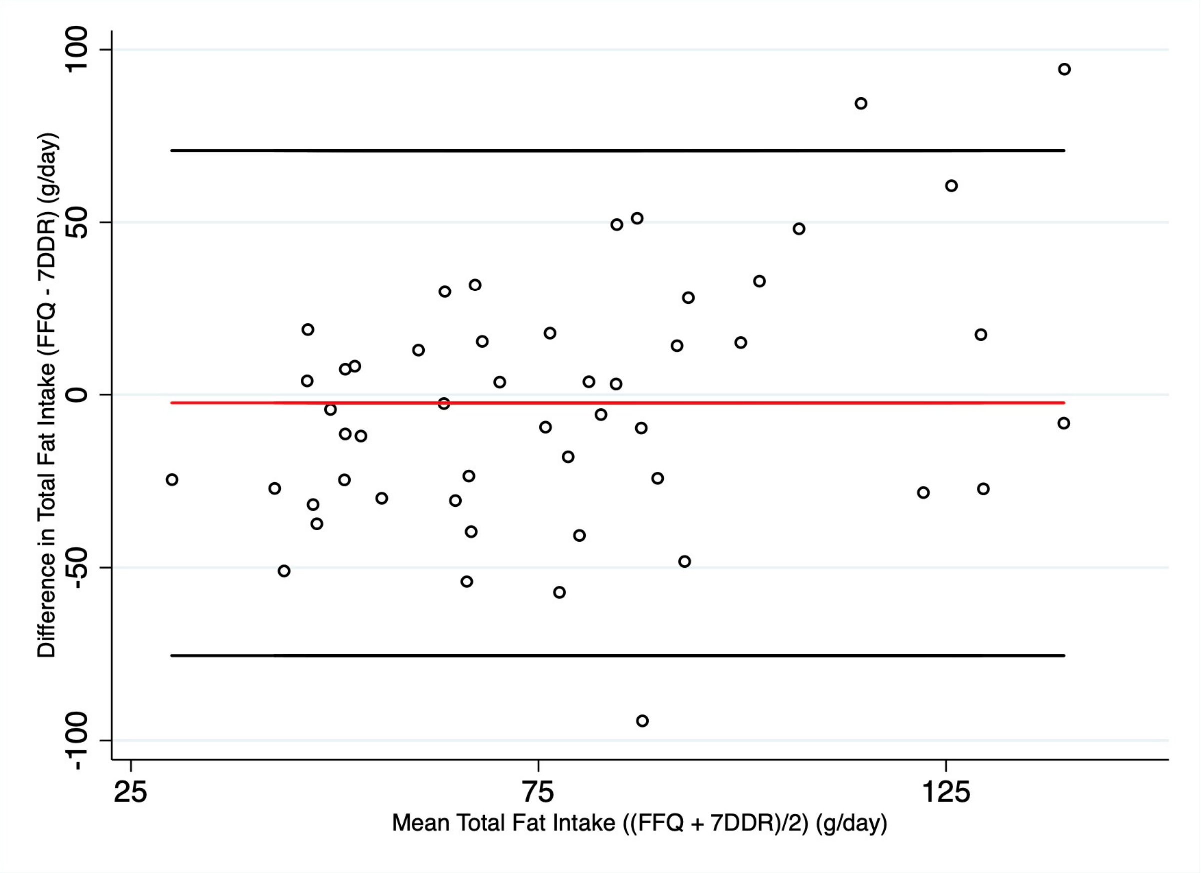

Figure S4: Mean differences of the total fat intake (g/day) derived from the FFQ and 7DDR (n=50). Solid black lines are the upper and lower 95% LOA -75.5 to 70.8). Red line is the mean difference (-2.38).

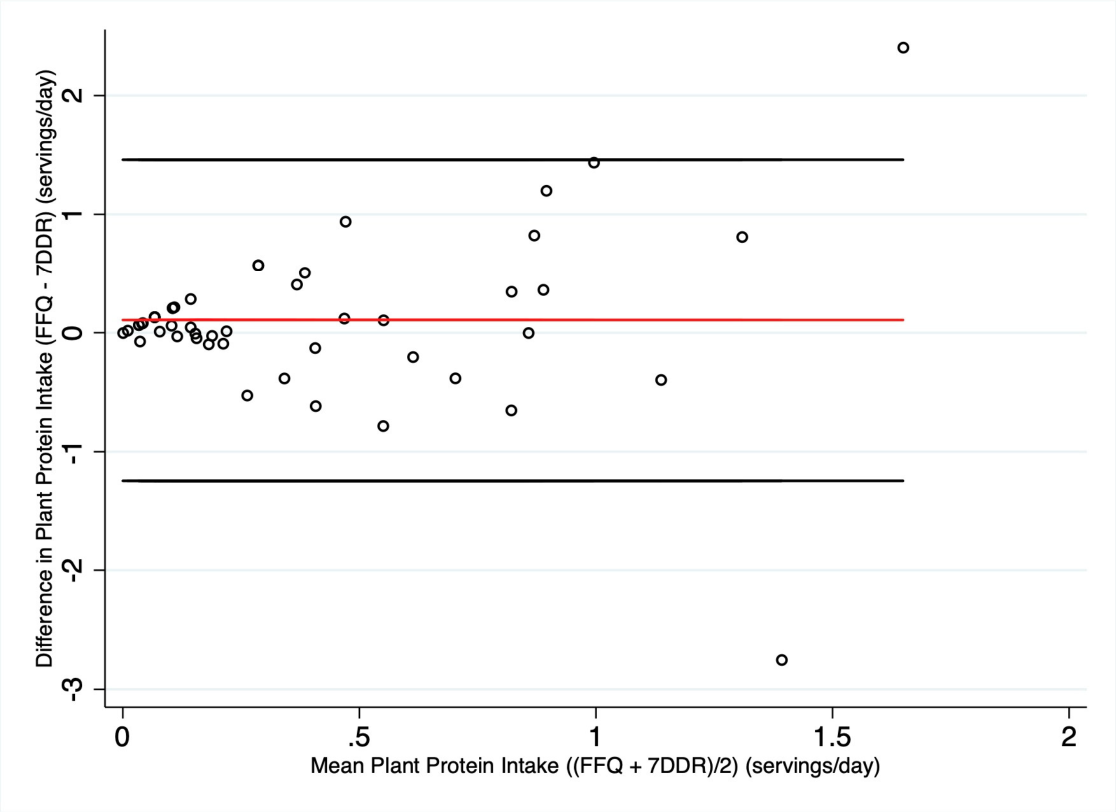

Figure S5: Mean differences of plant protein (servings/day) derived from the FFQ and 7DDR (n=50). Solid black lines are the upper and lower 95% LOA -1.25 to 1.46). Red line is the mean difference (0.11).

Intake of plant protein based on specific food items listed in Table 1.

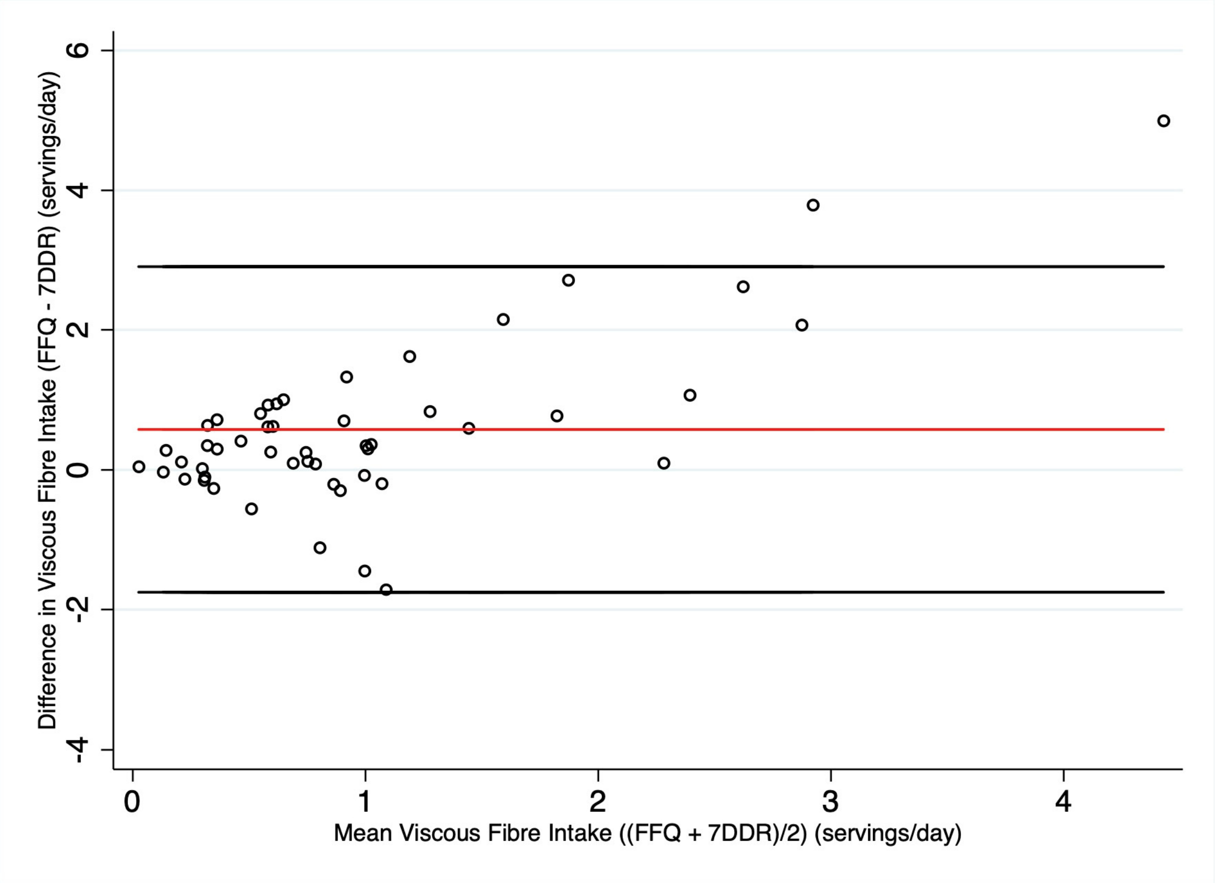

Figure S6: Mean differences of viscous fiber (servings/day) derived from the FFQ and 7DDR (n=50). Solid black lines are the upper and lower 95% LOA -1.75 to 2.91). Red line is the mean difference (0.58).

Intake of viscous fibre based on specific food items listed in Table 1.

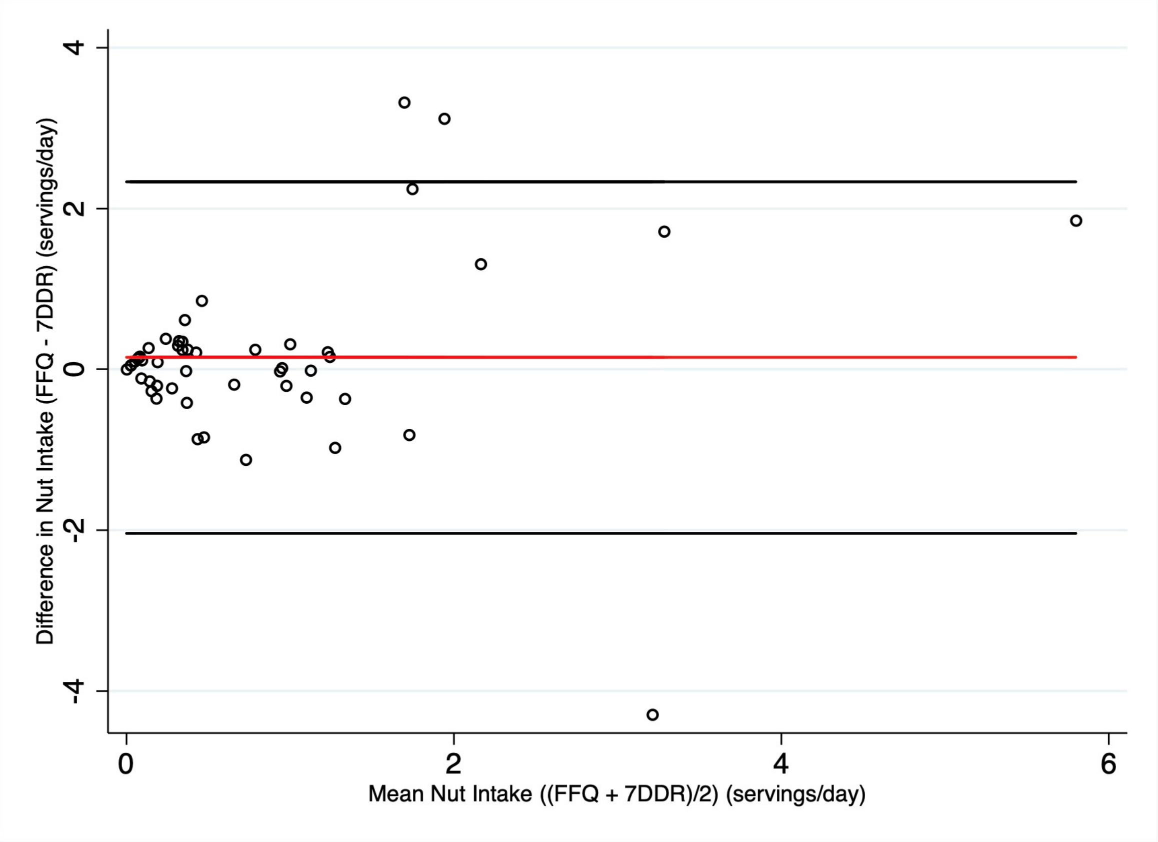

Figure S7: Mean differences of nuts (servings/day) derived from the FFQ and 7DDR (n=50).
Solid black lines are the upper and lower 95% LOA -2.04 to 2.33). Red line is the mean difference (0.15).

Intake of nuts based on specific food items listed in Table 1.

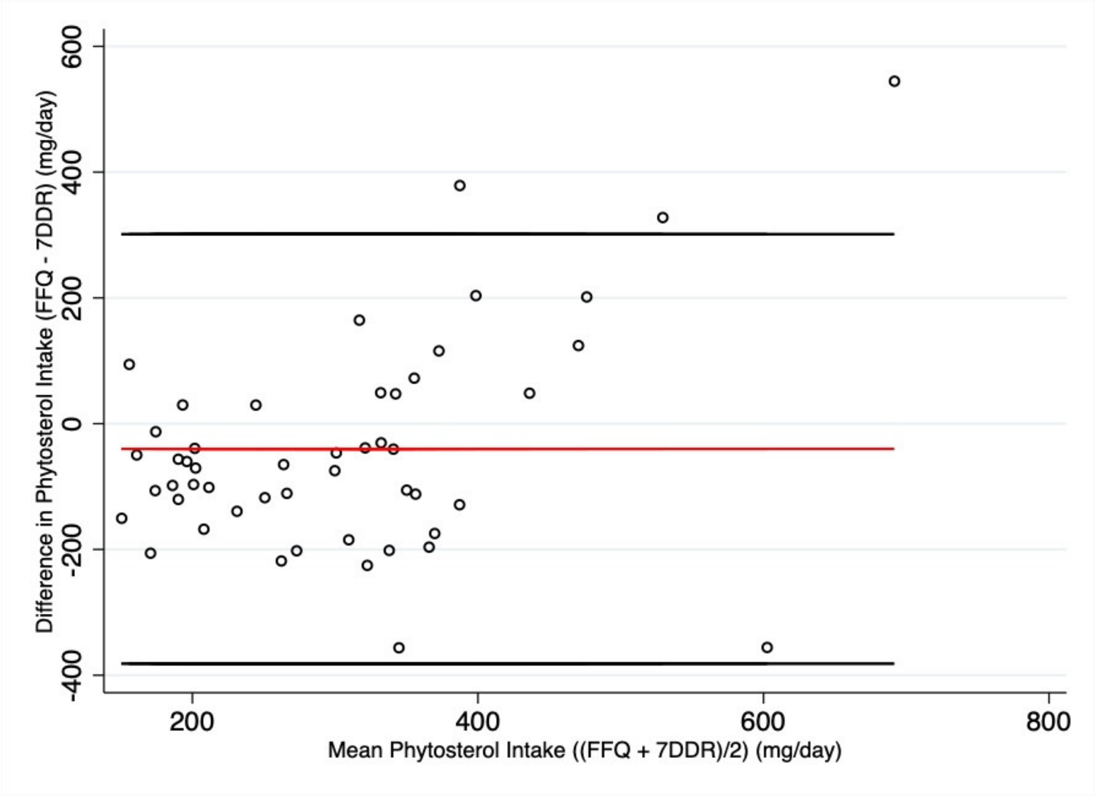

Figure S8: Mean differences of phytosterols (mg/day) derived from the FFQ and 7DDR (n=50).
Solid black lines are the upper and lower 95% LOA -381.6 to 301.4). Red line is the mean difference (-40.1).

Intake of phytosterols based on specific food items listed in Table 1.

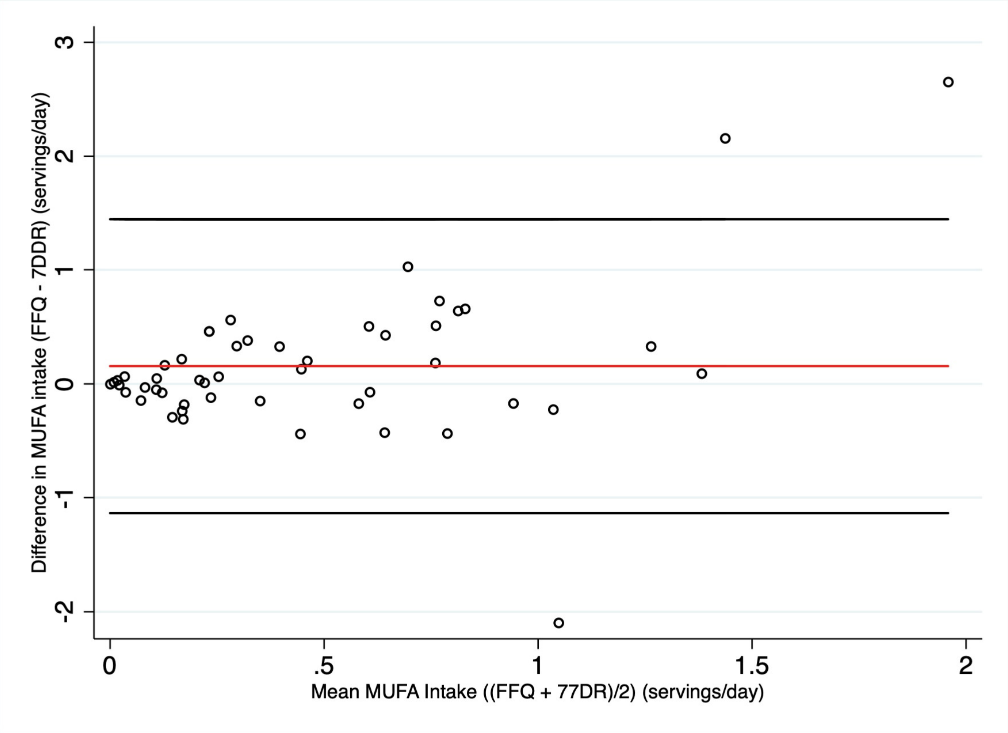

Figure S9: Mean differences of MUFAs (servings/day) derived from the FFQ and 7DDR (n=50). Solid black lines are the upper and lower 95% LOA -1.13 to 1.44). Red line is the mean difference (0.16).

Intake of MUFAs based on specific food items listed in Table 1.

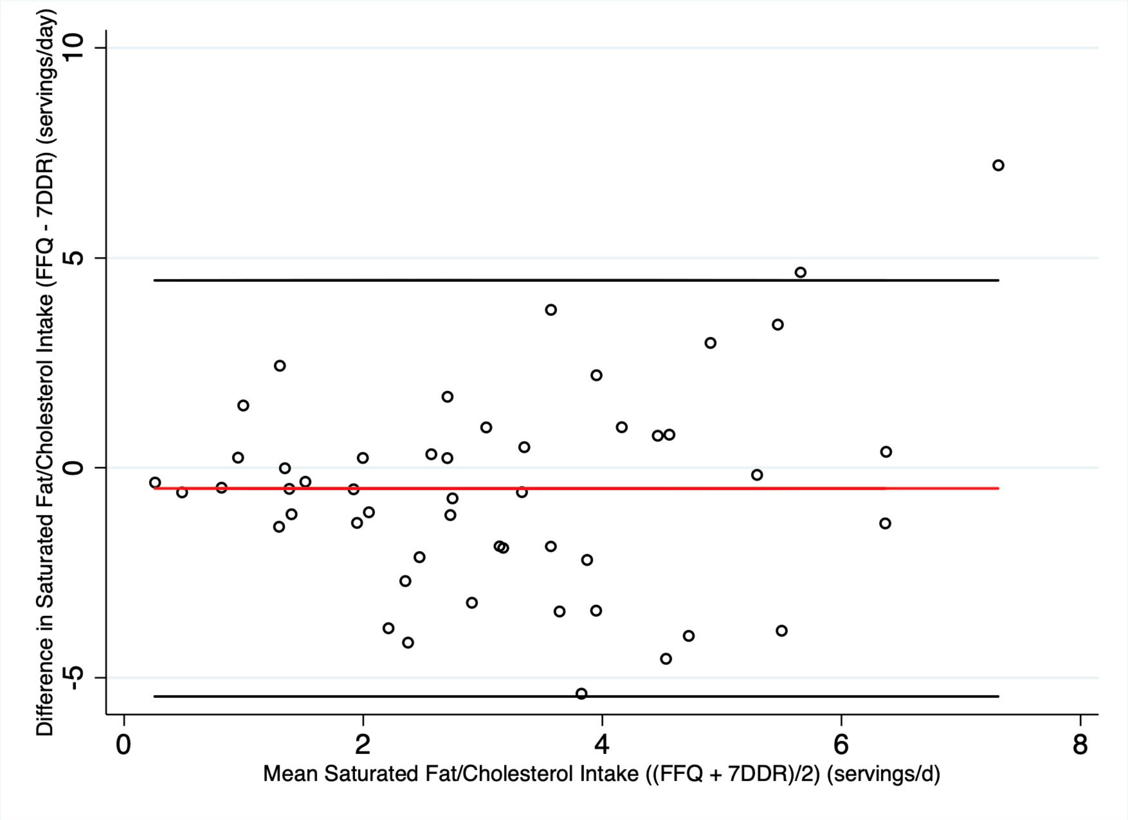

Figure S10: Mean differences of Saturated Fat/Cholesterol (servings/day) derived from the FFQ and 7DDR (n=50). Solid black lines are the upper and lower 95% LOA (-5.44 to 4.46). Red line is the mean difference (-0.50).

Intake of saturated fat/cholesterol based on specific food items listed in Table 1
